# IL-1β-driven amyloid plaque clearance is associated with an expansion of transcriptionally reprogrammed microglia

**DOI:** 10.1186/s12974-019-1645-7

**Published:** 2019-12-10

**Authors:** Fátima Rivera-Escalera, Jonathan J. Pinney, Laura Owlett, Hoda Ahmed, Juilee Thakar, John A. Olschowka, Michael R. Elliott, M. Kerry O’Banion

**Affiliations:** 1 Department of Neuroscience, University of Rochester School of Medicine and Dentistry, 601 Elmwood Avenue, Box 603, Rochester, NY 14642 USA; 2 David H. Smith Center for Vaccine Biology and Immunology, University of Rochester School of Medicine and Dentistry, Rochester, NY USA; 3 Department of Microbiology and Immunology, University of Rochester School of Medicine and Dentistry, Rochester, NY USA; 4 Del Monte Neuroscience Institute, University of Rochester School of Medicine and Dentistry, Rochester, NY USA

**Keywords:** Alzheimer’s disease, Interleukin-1β, Neuroinflammation, Microglia, Phagocytosis, Amyloid plaque, Proliferation, MX04

## Abstract

**Background:**

Neuroinflammation is thought to contribute to the pathogenesis of Alzheimer’s disease (AD), yet numerous studies have demonstrated a beneficial role for neuroinflammation in amyloid plaque clearance. We have previously shown that sustained expression of IL-1β in the hippocampus of APP/PS1 mice decreases amyloid plaque burden independent of recruited CCR2^+^ myeloid cells, suggesting resident microglia as the main phagocytic effectors of IL-1β-induced plaque clearance. To date, however, the mechanisms of IL-1β-induced plaque clearance remain poorly understood.

**Methods:**

To determine whether microglia are involved in IL-1β-induced plaque clearance, APP/PS1 mice induced to express mature human IL-1β in the hippocampus via adenoviral transduction were treated with the Aβ fluorescent probe methoxy-X04 (MX04) and microglial internalization of fibrillar Aβ (fAβ) was analyzed by flow cytometry and immunohistochemistry. To assess microglial proliferation, APP/PS1 mice transduced with IL-1β or control were injected intraperitoneally with BrdU and hippocampal tissue was analyzed by flow cytometry. RNAseq analysis was conducted on microglia FACS sorted from the hippocampus of control or IL-1β-treated APP/PS1 mice. These microglia were also sorted based on MX04 labeling (MX04^+^ and MX04^−^ microglia).

**Results:**

Resident microglia (CD45^lo^CD11b^+^) constituted > 70% of the MX04^+^ cells in both Phe- and IL-1β-treated conditions, and < 15% of MX04^+^ cells were recruited myeloid cells (CD45^hi^CD11b^+^). However, IL-1β treatment did not augment the percentage of MX04^+^ microglia nor the quantity of fAβ internalized by individual microglia. Instead, IL-1β increased the total number of MX04^+^ microglia in the hippocampus due to IL-1β-induced proliferation. In addition, transcriptomic analyses revealed that IL-1β treatment was associated with large-scale changes in the expression of genes related to immune responses, proliferation, and cytokine signaling.

**Conclusions:**

These studies show that IL-1β overexpression early in amyloid pathogenesis induces a change in the microglial gene expression profile and an expansion of microglial cells that facilitates Aβ plaque clearance.

## Introduction

Neuroinflammation has long been recognized as a key component in Alzheimer’s disease (AD) pathogenesis. However, several studies from our laboratory and others have shown that immune activation by proinflammatory cytokines (i.e., IL-1β, TNF-α, IL-6) leads to reductions in amyloid plaque burden [1–5]. In particular, we found that sustained hippocampal IL-1β expression decreases amyloid plaque accumulation in APPswe/PS1dE9 (APP/PS1) mice [4, 6] and in the 3xTgAD mouse model [7]. In contrast, several studies have shown that immune activation by anti-inflammatory cytokines (i.e., IL-10, IL-4) is associated with exacerbation of amyloid pathology [8–10], though other studies show beneficial effects [11–13]. These results indicate that the outcome of innate immune modulation in AD is extremely complex and still poorly understood.

Microglia are brain-resident innate immune effector cells and are widely thought to be the key mediators of amyloid beta clearance [14–16]. Microglial phagocytosis is believed to be dysfunctional in AD [17–19]. This impairment has been shown to correlate with increased production of proinflammatory cytokines and overwhelming accumulation of Aβ plaques in the AD brain [20–22]. However, several studies have shown that defects in the phagocytic function of microglia can be restored with Aβ immunization [18, 23] and anti-inflammatory drug treatment [24, 25]. Strikingly, activation of microglial cells by inflammatory molecules has been shown to enhance Aβ clearance by microglia [3, 26, 27]. Based on these studies, we reasoned that similarly, IL-1β might enhance Aβ clearance by modulating microglial cells. Our efforts to understand the mechanism(s) by which IL-1β mediates Aβ plaque clearance have focused on investigating the roles of resident microglia and recruited CCR2^+^ monocytes. Previously we showed that CCR2^+^ mononuclear cells are recruited to the inflamed hippocampus and associate with amyloid plaques in a CCR2-dependent manner [28]. However, disrupting CCR2 signaling and recruitment of BMD-mononuclear phagocytes did not alter the ability of IL-1β to reduce amyloid plaque burden [28].

In this study, we conducted in vivo phagocytosis assays and RNA profiling analysis of microglial cells to determine if IL-1β modulates the phagocytic clearance of fAβ by microglia. Our results showed that although microglia are activated, proliferate, and contribute to fAβ removal in the presence of IL-1β, we did not find clear evidence that IL-1β specifically enhanced the ability of microglia to phagocytose fAβ. Instead, we found that IL-1β significantly upregulated genes involved in immune responses, cytokine signaling, and proliferation. Our data point to a new mechanistic role for IL-1β in enhancing the number of microglia that are available to clear fAβ.

## Materials and methods

### Animals

Heterozygous APPswe/PS1dE9 (APP/PS1) mice on a congenic C57BL/6 background were purchased from the Jackson Laboratory (MMRRC stock #34832) and bred in-house under standard group housing conditions (*n* = 3–5 same-sex mice per cage). Both female and male mice were used to balance sex across our experimental conditions, and we tried to maintain equal numbers of female and male mice in all of our experiments. All animal procedures were reviewed and approved by the University of Rochester Committee on Animal Resources for compliance with federal regulations before the initiation of this study.

### Construction of recombinant adeno-associated virus serotype 2

The construction and characterization of rAAV2 has been previously described [28, 29]. The final plasmid containing a CMV promoter, an ssIL-1β construct that links the signal sequence of human IL-1ra to the mature form of IL-1β cDNA [30], producing a mature and secreted hIL-1β that does not require caspase-1 cleavage [29, 31], an SV40 polyA tail, and inverted terminal repeats, was used to produce recombinant adeno-associated virus serotype 2 using a baculovirus intermediary and S9 cells as previously described [32]. rAAV2-Phe-scFv was used as an irrelevant control viral vector; -Phe expresses a single-chain antibody against Phenobarbital [33]. AAV2 selectively transduces neurons and its use in the CNS is well characterized [34–36].

### Stereotactic injections

7.5 month-old APP/PS1 mice were anesthetized with 1.75% isoflurane, in 30% oxygen and 70% nitrogen and secured in a Kopf stereotactic apparatus using ear bars and a head holder. Ophthalmic ointment was applied to prevent drying of the eyes. The scalp was disinfected with betadine prior to incision with a scalpel. Two 0.5-mm burr holes were drilled, one on each side, at AP: − 2.06 and ML: ± 1.5 mm relative to bregma, and a 33 gauge needle attached to a 10 μl syringe (Hamilton, Reno, NV) was lowered 1.5 mm from the dural surface over 2 min. A Micro-1 microsyringe pump controller (World Precision Instruments) injected 5 μl of rAAV2-IL-1β or rAAV2-Phe using the convection-enhanced delivery method (CED), resulting in delivery of approximately 7.5 × 10^5^ infectious particles into each hippocampus as previously described [28]. Following rAAV2 delivery, 2 min was allowed for diffusion of viral particles. The needle was then raised over 2 min and the burr hole was sealed with bone wax. The procedure was then repeated to deliver the same viral vector on the opposite side. The scalp incision was closed with tissue adhesive (Vetbond). Betadine and topical lidocaine were applied to the top of the suture to prevent infection and for analgesia, respectively. Mice recovered in a heated area before being placed in their home cage. All animals were sacrificed 3–4 weeks post-viral transduction for brain tissue analysis. For assessment of BrdU+ microglia around amyloid plaques (Fig. 5c), APP/PS1 mice received bromodeoxyuridine (150 mg/kg) injections for three consecutive days prior to sacrifice to label proliferating cells.

### Immunohistochemistry

APP/PS1 mice were anesthetized with ketamine and xylazine and perfused with 0.15 M phosphate buffer (PB) containing 2 IU/ml heparin and 0.5% w/v sodium nitrite. The right half brain was fixed in ice-cold 4% paraformaldehyde (PFA), and the left hippocampus was dissected, snap-frozen in isopentane and stored at − 80 °C until further processing. The fixed half brain remained overnight in 4% PFA at 4 °C and was then transferred to 30% sucrose in 0.15 M PB until equilibrated. Brains were sectioned at 30 μm on a sliding microtome and free-floating sections stored in cryoprotectant until analyzed. Sections were washed in 0.15 M PB, blocked with 3% donkey normal serum (Sigma-Aldrich), and incubated in biotinylated mouse anti-6E10 (Covance, clone 6E10, 1:3000), rabbit anti-ionized calcium-binding adaptor molecule 1 (Iba-1; Wako Chemicals, 1:3000), rat anti-BrdU (Abcam, 1:300), or rabbit anti-glial fibrillary acidic protein (GFAP; Dako, 1:1000) for 48 h. For BrdU staining, sections were preincubated in 4 N hydrochloric acid for antigen retrieval. Antibody binding was visualized using secondary antibodies bound to Alexa 488, 594, or 647 fluorophores (Invitrogen, 1:2000), or Streptavidin 488 or 594 (Invitrogen, 1:1000).

### Image capture and analysis

Confocal images were obtained using an Olympus FV1000 laser scanning confocal microscope (Center Valley, PA) in the Confocal and Conventional Microscopy Core of the University of Rochester Medical Center Core Facility Program. All images were acquired using sequential scanning and oversaturation was prevented by using the hi-lo feature of the FV1000 software. UPLAN objectives were used to acquire the images. Cell counts in Figs. 2 and 3 were acquired from confocal z-stacks taken at 60× magnification. 3–5 images of the hippocampus were taken per section and 3 sections were analyzed per mouse. Microglia were determined based on Iba1 positivity and Hoechst nuclear stain was used to identify individual Iba1^+^ cells, which were manually counted by a blinded investigator. Only Iba1^+^ cells whose nuclei were present in the Z-stack were included in the analysis. Plaque-associated microglia were counted as previously described [4, 7]. In brief, the number of plaque-associated microglia was determined by counting Iba1^+^ cells with nuclei directly contacting 6E10^+^ amyloid plaques. BrdU^+^ microglia associated with plaques were imaged at 40×. The center of the plaque was selected and a circle with a 20-μm radius was drawn around the plaque. BrdU^+^Iba1^+^ cells whose cell bodies fell inside or within the 20-μm circle were included in the analysis.

### Flow cytometry

Three days prior to sacrifice, APP/PS1 mice received an intraperitoneal (i.p.) injection of methoxy-X04 (MX04) (10 mg/kg, Tocris) to label fibrillar amyloid beta plaques [37]. Brains were removed from transcardially-perfused mice, hippocampi dissected, and hippocampal tissue was homogenized using a Dounce homogenizer. Myelin was removed by magnetic separation using myelin depletion beads and LS columns (Miltenyi Biotec) according to the manufacturer’s protocol. Following myelin removal, cells were washed with FACS buffer (1× PBS containing 0.05% BSA), incubated in Fc block (BioLegend, clone 93, 1:100), and stained with CD11b-Alexa Fluor 488 (BD Pharmingen, clone M1/70, 1:200) and CD45-APC (BD Pharmingen, clone 30F11, 1:400). Propidium iodide (PI) was used as a viability marker. For the BrdU experiment (Fig. 5), APP/PS1 mice received bromodeoxyuridine (150 mg/kg) injections on Day 10th, Day 13th, and Day 16th post-rAAV2 transductions to label proliferating cells. Cells were stained with Fixable Viability Stain (FVS) 510 (BD Pharmingen) followed by staining of cell surface markers as described above. Following surface staining, cells were fixed and permeabilized for BrdU detection using the FITC BrdU Flow Kit (BD Pharmingen) following the manufacturer’s instructions. 50 μl of AccuCount Beads (Spherotech) was added to each sample to calculate the absolute number of cells in each sample. For cell sorting, hippocampal tissue was digested at 37 °C using the Neural Dissociation Kit (Miltenyi Biotec) prior to myelin removal. Following enzyme digestion, samples were kept at 4 °C until sorting, and microglial sorting was done at 4 °C. Samples were analyzed on a FACS LSRII (Becton Dickinson) or sorted on a FACSAria (Becton Dickinson) in the University of Rochester Medical Center Flow Cytometry Core facility and data was acquired using FlowJo v9 for Mac.

### Deep sequencing (RNA-seq) and data analysis

Sorted microglia were collected in 500 μl RLT Buffer (Qiagen) and total RNA was isolated using the RNeasy Mini Plus Kit (Qiagen). Low-input RNA-seq (Clontech SMARTer Technology) was performed by the University of Rochester Genomics Research Center. Briefly, 1 ng of total RNA was preamplified with the SMARter Ultra Low Input kit V2 (Clontech, Mountain View, CA). Libraries were constructed using the NexteraXT library kit (Illumina, San Diego, CA) and sequenced on the Illumina HiSeq2500 to generate approximately 20 million 100-bp single end-reads per sample. Two biological replicates of pooled cells from 6 to 8 APP/PS1 mouse hippocampi were sequenced for each experimental group.

RNA-Seq bam file was mapped to mm10 reference genome obtained from Ensemble. The data was analyzed using Rsamtools and GenomicAlignments [38]. GenomicAlignments count function summarizeOverlaps was used to generate the raw count data. The Bioconductor package DESeq2 was used to perform differential sequence analysis. The pairwise differential sequence analysis of raw count data from APP/PS1-Phe MX04^+^, APP/PS1-Phe MX04^−^, APP/PS1-IL-1β MX04^+^, and APP/PS1-IL-1β MX04^−^ mouse groups was performed. Genes were considered differentially expressed if q-value (*p* value corrected for multiple testing) < 0.05 and had a log2 |fold change| ≥ 1. plotPCA function was done using log transformed dataset from DESeq2. Venn diagram was generated using the VennDiagram package to look at intersections of DEGs lists pulled from comparisons made in DESeq2. Gene ontology (GO) terms from DEGs were obtained from the dataset using GoTermFinder and summarized using Revigo (http://revigo.irb.hr) [39]. Graph was exported from Revigo and manually curated in RStudio. All RNA-sequencing data files were submitted to the Gene Expression Omnibus (GEO) database under accession number GSE113539.

### qRT-PCR

Hippocampal tissue was homogenized using an Omni Tissue Homogenizer (Omni) and RNA was isolated using the RNeasy Mini Kit (Qiagen). One microgram of total RNA was reversed transcribed using the Superscript III First-Strand Kit (Invitrogen). PCR reactions were carried out in a final volume of 20 μl reactions containing iQ Supermix (Bio-Rad) and Applied Biosystems Taqman Kits for *Csf1* (Mm00432686_m1), *Il34* (Mm01243248_m1), *Tgfb1* (Mm01178820_m1), and *Il1b* (Mm00434228_m1). For *Gapdh*, the following sequences were used: forward, 5′ CCC AAT GTG TCC GTC GTG 3′; reverse, 5′ CCT GCT TCA CCA CCT TCT TG 3′; probe, 5′ TGT CAT CAT ACT TGG CAG GTT TCT CCA GG 3′. Samples were denatured at 95 °C for 3 min, followed by 40 cycles of denaturing at 95 °C for 30 s, annealing at 60 °C for 30 s and extension at 72 °C for 30 s. To determine relative difference in mRNA levels, reaction efficiency (*E*) was calculated from a standard curve and threshold cycle (*C*_t_) values were transformed using (1 + *E*)^*C*t^. GAPDH was used as a housekeeping to normalize the calculated quantities of mRNA for the gene of interest.

### Statistical analyses

Data was analyzed in Prism (GraphPad Software, San Diego, CA, USA) using unpaired *t* tests, multiple *t* tests, or two-way ANOVA analyses. Tukey’s multiple-comparisons test was used to further analyze significant ANOVAs. Multiple t tests were corrected for multiple comparisons using the Holm-Sidak method. All results are expressed as mean ± SEM. A value of *p* < 0.05 was considered significant.

## Results

### Microglia are the principal cell type internalizing Aβ in the presence of IL-1β-mediated neuroinflammation

**Fig. 1:**
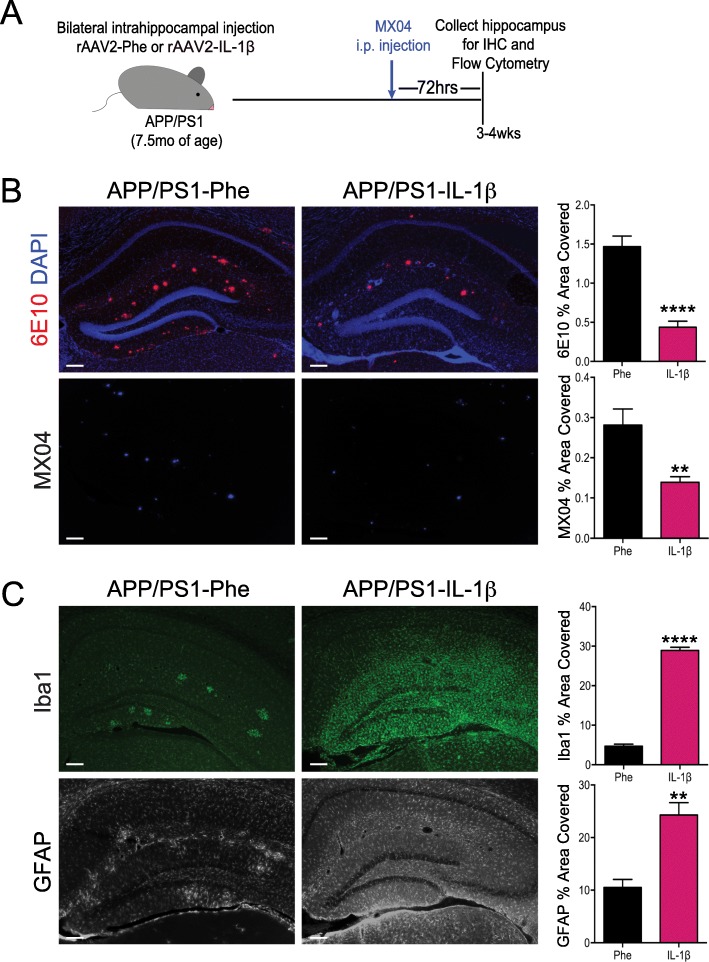
Sustained hippocampal expression of IL-1β reduces amyloid plaque burden in APP/PS1 mice. **a** Schematic of viral vector transduction and methoxy-X04 injections in APP/PS1 mice. APP/PS1 mice, aged 7–8 months, were treated with adenoviral vectors encoding cleaved, human IL-1β (rAAV2-IL-1β) or vector control (rAAV2-Phe) via intrahippocampal injection for 3–4 weeks. Three days prior to sacrifice and analysis, mice were given an i.p. injection of the fluorescent Aβ probe, methoxy-04 (MX04). **b** Representative images of 6E10 and MX04 IHC staining of amyloid plaques in APP/PS1 mice transduced with either rAAV2-Phe or rAAV2-IL-1β. Scale bar = 30 μm. Quantification of 6E10 (upper) and MX04 (lower) displayed as percent area of hippocampus covered by amyloid plaques. **c** Representative images of Iba1 and GFAP staining of microglia/macrophages and astrocytes, respectively in APP/PS1 mice transduced with either rAAV2-Phe or rAAV2-IL-1β. Scale bar = 30 μm. Percent area of Iba1 (upper) and GFAP (lower) staining in the hippocampus. *n* = 6 per group. Data displayed as mean ± SEM, unpaired *t* test, ****p < 0.0001, **p < 0.005

Because microglial phagocytosis of Aβ in the AD brain can be enhanced by other inflammatory challenges [27], we hypothesized that IL-1β could also modulate the clearance of fAβ by microglial cells. To test our hypothesis, APP/PS1 mice at 7.5 months of age were bilaterally transduced in the hippocampus with rAAV2-IL-1β (APP/PS1-IL-1β) or a control viral vector, rAAV2-Phe (APP/PS1-Phe), and sacrificed three to four weeks post-injection. Three days prior to sacrifice, mice received an i.p. injection of methoxy-X04 (MX04) to label fibrillar amyloid beta plaques (Fig. 1a). As previously described [28], APP/PS1 mice transduced with rAAV2-IL-1β showed significant reductions in hippocampal amyloid plaques compared to mice treated with rAAV2-Phe, as determined by immunohistochemical (IHC) analysis using both 6E10 staining and MX04 (Fig. 1b), and IL-1β reduced fibrillar and total amyloid plaque burden regardless of sex (Additional file 1). In addition, we found that pre-labeled MX04^+^ amyloid plaques were significantly decreased following IL-1β overexpression, suggesting that IL-1β drives reductions in existing amyloid plaques (see Additional file 2). This is consistent with our previously published data showing that IL-1β does not influence Aβ production [4, 6, 7, 28]. As expected, rAAV2-IL-1β transduction also induced microglial activation and astrogliosis, as indicated by staining for Iba1 and GFAP (Fig. 1c).

**Fig. 2:**
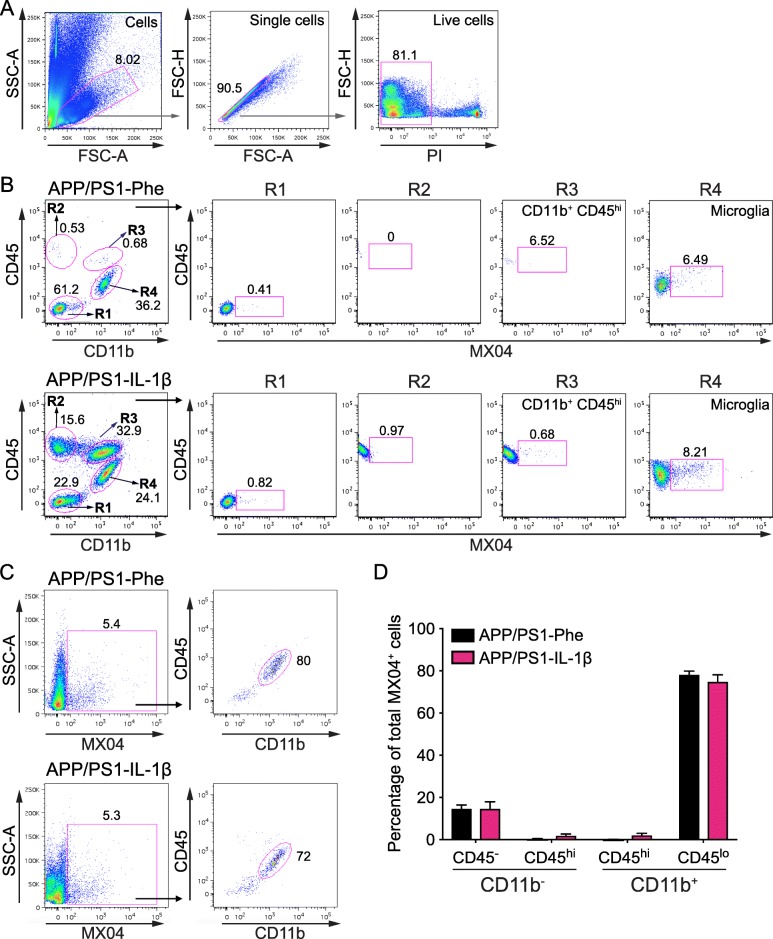
Microglia are the principal cell type internalizing Aβ in the absence and presence of IL-1β-mediated neuroinflammation. **a** Representative flow plots of the gating strategy utilized to analyze viable single cells isolated from mouse hippocampus. **b** Representative flow plots of the four populations that can be distinguished based on their differential expression of CD45 and CD11b, and representative flow plots of MX04^+^ cells in each population in the hippocampus of APP/PS1 mice transduced with rAAV2-Phe (top) or rAAV2-IL-1β (bottom). **c** Representative flow plots showing the distribution of MX04^+^ cells in the hippocampus of mice treated with rAAV2-Phe (upper) and rAAV2- IL-1β (lower). **d** Quantification of results in *C* for *n* = 9–10 per group. Data displayed as mean ± SEM, multiple *t* tests corrected for multiple comparisons using the Holm-Sidak method

We have previously demonstrated that expression of IL-1β in the hippocampus induces recruitment of CCR2^+^ monocytes, but these monocytes are not required for IL-1β-mediated plaque clearance [28]. However, our previous studies did not evaluate whether recruited phagocytes were capable of internalizing fAβ. To establish the contribution of different phagocytic cell populations in the clearance of Aβ following rAAV2-IL-1β transduction, single cell preparations of hippocampal tissue were subjected to flow cytometry analysis to identify MX04^+^ cells. We distinguished four cell populations based on their expression of CD45 and CD11b after gating to exclude debris, cell doublets, and dead cells (Fig. 2a, b). CD45 is differentially expressed on microglia (CD45^lo^/CD11b^+^) and recruited myeloid cells (CD45^hi^/CD11b^+^), and this distinction is commonly used in flow cytometry experiments to distinguish recruited myeloid cells from resident microglia in various disease states [27, 40, 41]. In agreement with our previous observations [28, 30] there was minimal recruitment of CD45^hi^ peripheral immune cells (CD11b^+^ and CD11b^−^) in APP/PS1-Phe mice, but these populations were significantly increased in the hippocampus following IL-1β overexpression (Fig. 2b, Additional files 3 and 4). We found that microglia are the principal cell type internalizing fAβ, regardless of the presence and absence of IL-1β overexpression (Fig. 2b, c). Moreover, we observed minimal uptake of MX04^+^/Aβ by non-microglia populations, including BMD-myeloid cells (CD45^hi^CD11b^+^), other recruited leukocytes (CD45^hi^CD11b^−^), and other brain resident cells (CD45^−^CD11b^−^) in the absence or presence of IL-1β treatment (Fig. 2b, c). Specifically, we found that resident microglia (CD45^lo^CD11b^+^) constituted > 70% of the MX04^+^ cells in both control and IL-1β-treated conditions, and that < 15% of MX04^+^ cells were recruited myeloid cells (CD45^hi^CD11b^+^) (Fig. 2d and Additional file 4).

### Effects of IL-1β treatment on the capacity of microglia to internalize Aβ

**Fig. 3:**
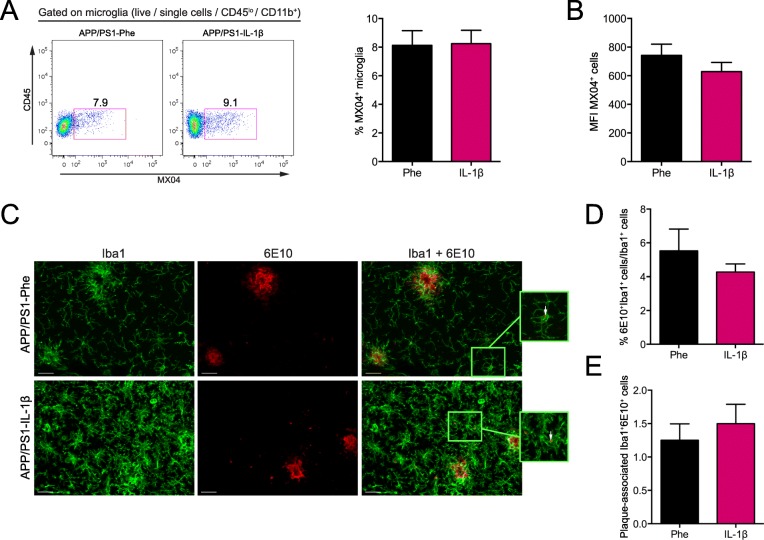
IL-1β does not alter the phagocytic capacity or localization of microglia to Aβ plaques in vivo. **a** Representative flow plots of hippocampal microglia (CD45^lo^CD11b^+^) that internalized MX04^+^-Aβ in the absence and presence of IL-1β overexpression (left), and percentage of MX04^+^ microglia (right). **b** Mean fluorescence intensity of MX04 signal in microglia for *n* = 10–12 per group. Data displayed as mean ± SEM, unpaired *t* test. **c** Representative images of Iba1 and 6E10 staining microglia and amyloid beta plaques in the hippocampus of APP/PS1 mice transduced with either rAAV2-Phe or rAAV2-IL-1β. Scale bar = 20 μm. **d**, **e** Quantification of the total number of Iba1^+^6E10^+^ cells per mm^2^ (*D*), % of 6E10^+^Iba1^+^/Iba1^+^ cells (*E*), and the number of plaque-associated Iba1^+^ cells that internalize 6E10^+^-Aβ (right). *n* = 5 per group. Data displayed as mean ± SEM, unpaired *t* test

Having found that microglia were the predominant cell population engulfing fAβ regardless of IL-1β treatment, we asked whether plaque clearance induced by IL-1β was associated with an increase in the ability of microglia to internalize Aβ. Flow cytometry analysis revealed that approximately 8% of hippocampal microglia were MX04^+^ in both control- and IL-1β-treated mice (Fig. 3a), indicating that IL-1β does not increase the overall fraction of microglia actively engulfing fAβ. Using the mean fluorescence intensity (MFI) of MX04 to measure the total amount of fAβ internalized per microglia, there was no significant difference in the MX04 MFI between microglia in control versus IL-1β-treated mice (Fig. 3b). Consistent with these findings, IHC analyses showed that IL-1β did not affect the percentage of Aβ^+^ microglia (6E10^+^ Iba1^+^) in the hippocampus, nor did IL-1β affect localization of microglia that took up Aβ (6E10^+^ Iba1^+^) to Aβ plaques (Fig. 3c–e). Thus, the effects of IL-1β on plaque clearance in vivo did not appear to be associated with an increase in the proportion of resident microglia containing fAβ.

### IL-1β increased the total number of microglia that correlates with increased numbers of microglia with internalized Aβ

**Fig. 4:**
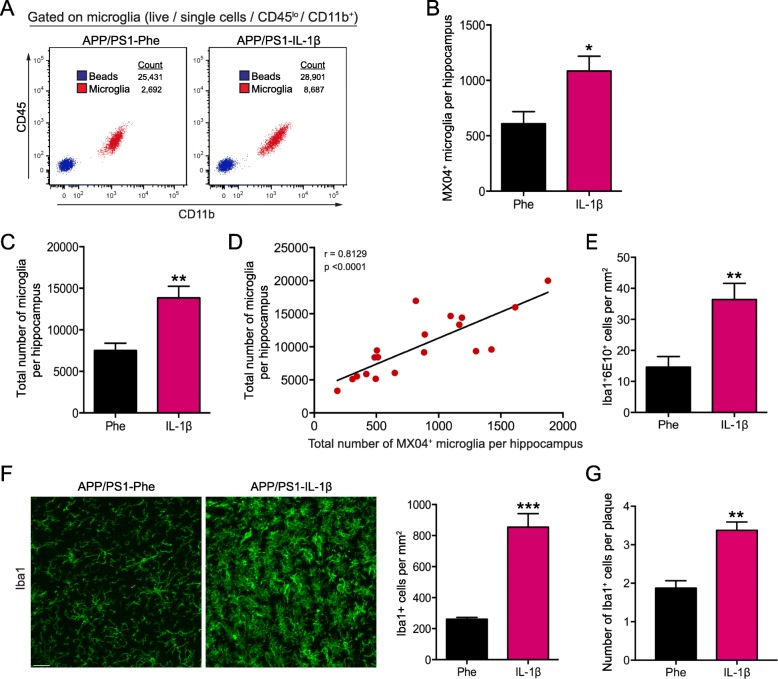
IL-1β increases the total number of microglia that correlates with the number of microglia that internalized Aβ. **a** Representative flow plots used for analysis of total microglia (CD45^lo^CD11b^+^) cell counts in the hippocampus of APP/PS1 mice transduced with either rAAV2-Phe or rAAV2-IL-1β. A fixed volume of AccuCount beads (blue) was added to each sample in order to calculate the total number of microglia (red) in each sample as described in Methods. **b** Number of MX04^+^ microglia per hippocampus in rAAV2-Phe or rAAV2-IL-1β treated mice. **c** Total number of microglia per hippocampus in rAAV2-Phe or rAAV2-IL-1β treated mice. *n* = 10–12 per group. Data displayed as mean ± SEM, unpaired *t* test, *p < 0.05, **p < 0.005. **d** Correlation between the total number of microglia and the total number of microglia that internalized MX04^+^-Aβ calculated using Pearson correlation coefficient. **e** The number of Iba1^+^6E10^+^ cells per mm^2^ in APP/PS1 mice transduced with rAAV2-Phe or rAAV2-IL-1β was determined from data shown in Figure 3C. **f** Representative images (left) and quantification (right) of total Iba1^+^ microglia, and **g** the number of plaque-associated Iba1^+^ microglia in APP/PS1 mice transduced with either rAAV2-Phe or rAAV2-IL-1β. Scale bar = 20 μm. *n* = 5 per group. Data displayed as mean ± SEM, unpaired *t* test, **p < 0.01, ***p < 0.0005

Having found that the total percentage of microglia internalizing fAβ did not change with IL-1β overexpression, we considered whether increased plaque clearance under these conditions was associated with a change in the overall number of MX04^+^ microglia in the hippocampus. To test this, we included quantitation beads in our flow cytometry samples and calculated the total number of MX04^+^ and MX04^−^ microglial cells under each treatment condition (Fig. 4a). We found that IL-1β significantly increased the total number of MX04^+^ microglial cells in the hippocampus of APP/PS1 mice by nearly two-fold (Fig. 4b), and this increase was associated with an increase in the total number of microglia per hippocampus (Fig. 4c, d). These findings were corroborated by IHC analysis of 6E10^+^ and Iba1^+^ cells per mm^2^ in the hippocampus of APP/PS1-Phe and APP/PS1-IL-1β mice, which revealed an IL-1β associated doubling of such cells (Fig. 4e, f). Total plaque-associated microglia were also increased following IL-1β overexpression (Fig. 4 g). Taken together, these findings suggest that IL-1β evoked increases in the total number of microglial cells, facilitating Aβ removal.

### IL-1β induces microglial proliferation

**Fig. 5:**
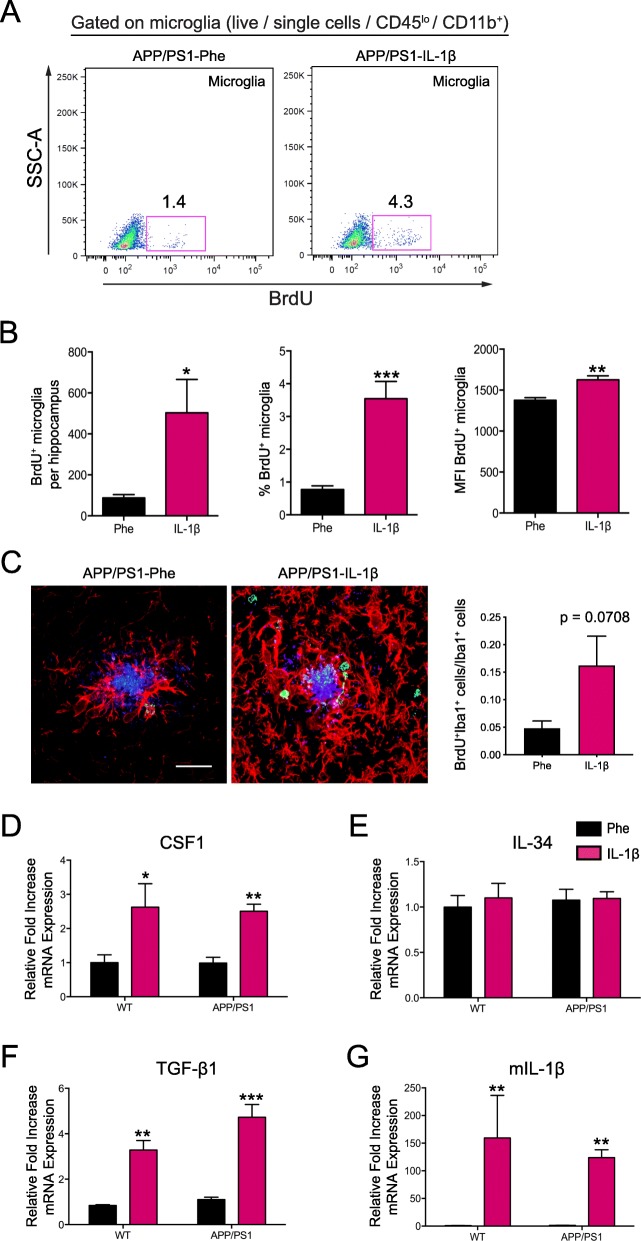
IL-1β overexpression induces proliferation of hippocampal microglia. **a** Representative flow plots of BrdU^+^ proliferating hippocampal microglia (CD45^lo^CD11b^+^) in the absence and presence of IL-1β overexpression. **b** Quantification of the total number of BrdU^+^ microglia (left), percentage of BrdU^+^ microglia, and the mean fluorescence intensity (MFI) of BrdU expression. *n* = 6 per group. Data displayed as mean ± SEM, unpaired *t* test, *p < 0.05, **p < 0.005, ***p < 0.0005. **c** Representative images (left) and quantification (right) of the proportion of BrdU^+^ (green) plaque-associated microglia (Iba1^+^; red). Scale bar = 20 μm. *n* = 5 per group. Data displayed as mean ± SEM, unpaired *t* test. **d**–**g** Quantitative real-time PCR of hippocampal tissue from APP/PS1 mice and age-matched wild-type controls transduced with either rAAV2-Phe or rAAV2-IL-1β showing mRNA expression relative to control for murine *Csf1r* (**c**), ***Il34*** (**d**)**,** *Tgfb1* (**e**), and *Il1b* (**f**). All samples were normalized to *Gapdh*. *n* = 6–12 per group. Data displayed as mean ± SEM, two-way ANOVA, *p < 0.05, **p < 0.005, ***p < 0.0005

It has been reported that increases in the resident microglial population under inflammatory conditions can be supported by local self-proliferation and that this proliferation depends on CSF1R signaling [42]. Thus, we asked whether IL-1β increases the total number of microglial cells in the hippocampus by inducing microglial proliferation. To address this hypothesis, APP/PS1-Phe and APP/PS1-IL-1β mice received an i.p. injection of BrdU (150 mg/kg) on days 10, 13, and 16 post-rAAV2 transductions to label proliferating cells and were then analyzed three weeks post-transduction. Flow cytometry revealed increases in the total number of proliferating microglia (BrdU^+^), the percentage of BrdU^+^ microglia, and the mean fluorescence intensity of BrdU expression in microglial cells in APP/PS1-IL-1β mice (Fig. 5a, b). Immunohistochemical analysis also revealed that the proportion of BrdU^+^ plaque-associated Iba1^+^ microglia was increased (Fig. 5c). These results indicate that IL-1β induces microglial proliferation that could be important for fAβ removal.

We next conducted quantitative RT-PCR to evaluate whether sustained IL-1β expression induces the expression of growth factors involved in microglial proliferation and/or survival. We focused on macrophage colony-stimulating factor-1 (CSF-1), IL-34, and transforming growth factor beta-1 (TGF-β1) because these cytokines have been shown to play important roles in microglial proliferation and/or survival [43, 44]. We found that CSF-1 and TGF-β1 mRNA levels were increased in the hippocampus of age-matched non-transgenic controls and APP/PS1 mice following IL-1β overexpression while the level of IL-34 mRNA was not altered (Fig. 5d–g). As expected, we also corroborated that human IL-1β overexpression induces an increase in the mRNA levels of murine IL-1β in both age-matched non-transgenic controls and APP/PS1 mice when compared to APP/PS1-Phe mice (Fig. 5 g). These studies indicate that IL-1β induces an environment that is suitable for sustaining microglial proliferation and survival.

### Minimal effects of IL-1β treatment on the expression of phagocytosis-related genes in microglia

**Fig. 6:**
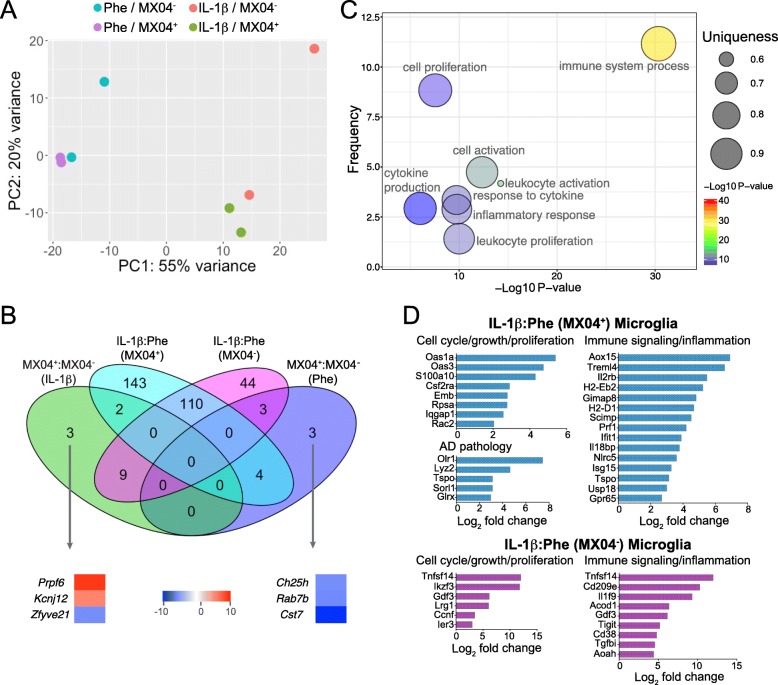
IL-1β, but not Aβ phagocytosis, is associated with large-scale shifts in hippocampal microglia gene expression. **a** Principal component analysis of 500 top variance genes. **b** Venn diagram illustrating intersecting differentially expressed genes between each of four tested conditions. **c** Scatterplot of summarized GO terms obtained using Revigo. Metrics plotted on the graph include relative frequency of the term in the reference gene database, -log10 of *p* value, and uniqueness of the term relative to other terms in the entire list. A subset of the summarized GO Terms is shown for easier visualization. **d** Genes of interest associated with IL-1β treatment in MX04^+^ and MX04^−^ microglia. Values plotted indicate log2 fold change from IL-1β-treated relative to Phe-treated in MX04^+^ (top) and MX04^−^ microglia (bottom). Two biological replicates of pooled cells from 6–8 APP/PS1 mouse hippocampi were sequenced for each experimental group

To better understand the effects of IL-1β signaling on microglia in AD, we conducted RNAseq analysis on microglia FACS-sorted from the hippocampus of control- or IL-1β-treated APP/PS1 mice. In addition, microglia were sorted based on MX04 fluorescence using the in vivo Aβ labeling method in Fig. 1a in order to determine whether microglia internalizing Aβ displayed a distinct gene expression profile compared to Aβ^−^ microglia. Analysis of differentially regulated genes (DEGs) from these groups revealed that IL-1β treatment was a much stronger driver of differential gene expression than was Aβ internalization (Fig. 6a, b). In fact, a direct comparison of DEGs among Aβ^+^ and Aβ^−^ showed a total of only 6 DEGs specific to Aβ^+^ microglia (Fig. 6b; Additional file 5). Of these genes, only two have been linked to phagocytosis, *Rab7b* and *Ch25h* [45, 46], and both of these were reduced in MX04^+^ compared to MX04^−^ microglia in control-treated mice (Fig. 6b).

### IL-1β treatment induces large-scale changes to microglia gene expression

Analysis of microglia gene expression revealed 315 DEGs in IL-1β-treated versus Phe-treated AD mice, of which 110 were shared between MX04^+^ and MX04^−^ microglia (Fig. 6b; Additional file 5). Of these genes, 44 were differentially expressed in MX04^−^ microglia between IL-1β and Phe conditions (Fig. 6b), and 143 genes were altered in MX04^+^ microglia between IL-1β and Phe conditions (Fig. 6b). To better understand the effects of IL-1β on microglia in AD, we summarized gene ontology (GO) terms obtained from the dataset using GoTermFinder then summarized the GO terms using Revigo [39] to identify significant broad alterations in microglia cellular processes induced by IL-1β. These results showed that the cellular processes most impacted by IL-1β treatment were immune responses, proliferation and cytokine signaling (Fig. 6c).

In MX04^−^ microglia, IL-1β increased the expression of genes involved in immune signaling and inflammation (*Cd38, Gdf3, CD209e, Tnfsf14, Acod1*, *Il1f9, Tigit, Tgfbi, Aoah*), and cell cycle and proliferation (*Ccnf, Tnfsf14, Ikzf3, Ier3*, *Gdf3*, *Lrg1*). In MX04^+^ microglia, IL-1β upregulated several genes involved in the regulation of cell proliferation, growth, differentiation, and cell cycle progression (*Csf2ra, Oas1a, Oas3, Rpsa, S100a10, Emb, Iqgap1, Rac2*), and immune signaling (*Gpr65, Usp18, Alox15, Il2rb*, *Isg15, Ifit1, Prf1, Il18bp, Tspo, Gimap8, H2-Eb2, H2-D1, Scimp, Treml4, Nlrc5*) (Fig. 6d). IL-1β also upregulated several genes involved in AD pathology such as *Lyz2*, *Sorl1, Olr1, Tspo,* and *Glrx* in MX04^+^ microglia (Fig. 6d).

## Discussion

In this study, we utilized flow cytometry, immunohistochemistry, and RNAseq to investigate whether IL-1β modulates the clearance of fibrillar Aβ by hippocampal microglial cells. We found that microglial cells are the principal cell type internalizing fAβ under all conditions, and that IL-1β did not appear to modulate the ability of microglial cells to clear fAβ. Instead, IL-1β increased the total number of microglial cells by inducing microglial proliferation. In addition, RNA profiling of microglial cells revealed that IL-1β overexpression upregulates genes involved in immune responses, cytokine signaling, and proliferation that may be related to Aβ clearance.

Microglia associate with Aβ plaques and express high levels of proinflammatory molecules [47]. Because microglial activation by other inflammatory cytokines can lead to Aβ clearance in vivo [3, 16, 27], we hypothesized that IL-1β overexpression creates an environment in which the resident microglial population is sufficiently activated to become better phagocytes. Our in vivo phagocytosis assays revealed that microglia represented the main cell type internalizing Aβ in control-treated and IL-1β-treated mice. However, although our studies showed that IL-1β increased the total number of microglia that internalized fAβ, we did not find evidence that IL-1β enhanced the ability of individual microglial cells to engulf fAβ. These findings are consistent with a study that found no change in Aβ phagocytosis in microglia producing the cytokine IL-1β [20]. Although our results are most consistent with the interpretation that increased microglia number is the mechanism underlying reduced Aβ plaque load in our model of sustained IL-1β overexpression, we have not addressed the possibility that microglia in this environment increase their uptake and degradation (e.g. flux) of fAβ or take up soluble forms of Aβ that contribute to overall amyloid plaque burden. Studies using primary microglial cell cultures or ex vivo preparations of isolated microglia from IL-1β-treated mice provide possible approaches to explore the dynamics of Aβ uptake and degradation; however, they do not recapitulate the complex signaling within the inflamed CNS. Moreover, translating findings in these models to the in vivo situation is further hampered by substantial changes in cell phenotype and gene expression brought about by culture and isolation techniques [43]. Thus, a better approach to examine the dynamics of fAβ handling by microglia would be the use of live two-photon imaging, which is beyond the scope of the current work.

Notably, we did not find that the % of MX04^+^ recruited myeloid cells significantly increased in IL-1β-treated mice. This is in agreement with our previous studies showing that recruited myeloid cells are not essential for IL-1β-mediated fibrillar Aβ clearance [28], and consistent with several studies showing that recruited myeloid cells are not as competent at restricting fAβ as resident microglia [48–50]. Because our study focused on fAβ, we cannot rule out the contribution of recruited myeloid cells in removing soluble Aβ in our model. In fact, studies have shown that a decrease in CCR2^+^ myeloid cells in APP/PS1 mice at 6 months of age was associated with increased levels of soluble oligomeric Aβ in the hippocampus and memory impairments [51]. One caveat of our flow cytometry analysis is that it did not distinguish between different CD45^hi^ myeloid and lymphoid populations, or other brain resident populations such as astrocytes. Previous work from our lab has shown that IL-1β induces significant recruitment of neutrophils [30], and neutrophils have been shown to modulate Alzheimer’s disease pathology [52, 53]. Likewise, our data suggests that IL-1β induces recruitment of lymphocytes (CD45^hi^CD11b^lo^) (Fig. 2b, Additional file 3) and we have previously shown that IL-1β induces recruitment of CD4^+^ and CD8^+^ T cells [30]. IL-1β increases IL-4 production, a potent modulator of CD4 T cell responses, and administration of IL-4 in AD mice induces amyloid plaque clearance [11]. Importantly, Marsh and colleagues [54] showed that lack of T cells, B cells, and natural killer cells in a mouse model of AD leads to exacerbation of amyloid pathology, and this was associated with altered microglial function. Taken together these studies suggest that there is cross-talk between microglia and T cells, and that adaptive immune cells can modulate microglial function. IL-1β overexpression also induces astrogliosis (Fig. 1c), and astrocytes have been shown to restrict amyloid pathology [55–57]. Moreover, astrocytes can also modulate microglial responses to Aβ [55, 58, 59]. Thus, future studies are required to address possible roles for other CD45^hi^ leukocytes and astrocytes in facilitating Aβ plaque removal. Given known regional differences in microglial populations, future studies will also be required to address whether the effects of IL-1β on microglial responses and plaque clearance are replicated in other brain regions such as the cerebral cortex [60].

Microglial proliferation has been shown to be the main contributor to increased microglial numbers under certain disease states such as ischemia, facial nerve transection, prion disease, and Alzheimer’s disease [42, 61–64]. The role of microglial proliferation in AD is not fully understood. Human studies have shown that proliferating microglia are visible in the vicinity of amyloid plaques in the hippocampus [65] and throughout the temporal cortex of the AD brain [42, 63] suggesting that proliferating microglia may contribute to pathology in late-stage AD. Apart from proliferation, several studies have suggested that microglial activation plays a beneficial role in early stages of AD pathology, but contributes to disease worsening in late stages of AD [66–68]. Longitudinal PET imaging studies in AD patients showed that a high baseline level of microglial activation was associated with stable clinical progression in early disease stages while low microglial activation was associated with faster decline [69]. Furthermore, microglial activation in early stages of amyloid accumulation is associated with the preservation of hippocampal and gray matter volume [70]. Our studies in APP/PS1 mice give insight into the complexity of microglial activation and proliferation that are particularly relevant to early amyloid pathogenesis. Specifically, our studies show that IL-1β overexpression early in amyloid pathogenesis induces a change in the microglial gene expression profile and an expansion of microglial cells that facilitates Aβ removal.

Microglial proliferation and survival have been attributed to activation of CSF1R receptor on microglia by its two main ligands CSF-1 and IL-34 [42]. APP/PS1 mice treated with CSF-1 or IL-34 show increased numbers of microglial cells and increased Aβ clearance [71, 72]. CSF-1 treatment has been shown to improve memory deficits and ameliorate amyloid plaque burden in the APP/PS1 mice [71]. However, CSF-1 treatment in the hAPP mouse model of AD improved cognitive function without altering amyloid deposition [73]. Interestingly, inhibition of microglial proliferation by blocking CSF1R prevented behavioral deficits and synaptic degeneration, but did not alter amyloid pathology in APP/PS1 mice [63]. TGF-β1R is a microglial-specific gene, and microglia are absent in TGF-β1-deficient mice, indicating an important role of TGF-β1 signaling for microglial survival [43]. In addition, TGF-β1 overexpression leads to amelioration of amyloid pathology in a mouse model of AD [74].

Our studies showed that IL-1β increased the total number of microglial cells by inducing microglial proliferation, concomitant with increased levels of CSF-1 and TGF-β1. Microglial proliferation may therefore be an important step in facilitating Aβ removal during chronic IL-1β overexpression. In fact, several studies have shown increased microglial proliferation associated with enhanced amyloid plaque clearance [26, 71, 72, 75]. In our studies we attempted to determine if microglia that proliferated directly contributed to Aβ clearance. Unfortunately, the BrdU staining for flow cytometry was not compatible with the MX04 signal, thus we were not able to make any conclusions on Aβ internalization in BrdU^+^ microglial cells. Studies have shown that astrocytes can secrete CSF-1 and TGF-β1 in response to IL-1β treatment in vitro [76, 77]. Importantly, treatment of astrocyte-microglia co-cultures with IL-1β increased microglial proliferation, and treatment with anti-CSF-1 antibody prevented microglial proliferation [78]. Because our immunohistochemical analysis showed that astrocytes are significantly activated in APP/PS1 mice injected with AAV2-IL-1β (Fig. 1c), it is possible that astrocyte secretion of CSF-1 in our model may be facilitating microglial proliferation. Future studies will need to address if astrocytes modulate microglial proliferation in vivo in our model, and identify which cell types are the major sources of CSF-1 and TGF-β1.

RNAseq analysis revealed several genes related to cell proliferation, cell differentiation, cell cycle progression, and immune signaling that are expressed preferentially by MX04^+^ microglial cells. Of interest, IL-1β significantly enriched *Csf2ra* transcript in MX04^+^ microglia. Csf2ra is a receptor for GM-CSF, a growth factor that promotes survival and activation of several immune cells including macrophages [79], and is important in mediating tissue inflammation [80]. Csf2ra expression is increased on phagocytic microglia following gamma oscillation mediated amyloid plaque reductions in the 5xFAD mouse model of AD [81]. Interestingly, exposing old microglia from APP/PS1 mice to conditioned media of young WT microglia or treatment with GM-CSF induced microglial proliferation and a reduction in amyloid plaques [26]. Because our studies showed that IL-1β induced microglial proliferation and increased Csf2ra expression in MX04^+^ microglia, further investigation into the role of Csf2ra in IL-1β-mediated amyloid plaque clearance is warranted.

Recently, studies have described a microglia type associated with neurodegenerative diseases [82, 83]. In our studies, IL-1β upregulated *Lyz2,* a gene shown to be expressed on disease-associated microglia in a Trem2 independent manner [82]. In addition, IL-1β also increased the expression of other genes associated with AD pathology such as *Sorl1* and *Tspo* in MX04^+^ microglia. SORL1 is decreased in individuals with mild cognitive impairment [84] and in patients with AD [85, 86], and SORL1 deficiency has been linked to the development of sporadic AD [85, 87, 88]. Loss of SORL1 in the APP/PS1 mouse model of AD leads to early deposition of amyloid plaques [89], and SORL1 overexpression decreases amyloid beta concentrations in the PDAPP mouse model of AD [90]. Thus, SORL1 plays a protective role against AD [88–91]. Future studies should evaluate microglial expression of SORL1 and its contribution to amyloid beta clearance in the context of chronic neuroinflammation.

IL-1β also increased the expression of *Tspo* in MX04^+^ microglia. TSPO radiotracers have been used in positron emission tomography (PET) studies as a marker of microglial activation [92]. TSPO signal is increased in the brains of aged individuals and in patients with AD [69, 92–94]. In agreement, TSPO expression is increased in the 5XFAD mouse model of AD, and TSPO is expressed mainly by microglia [93]. Administration of the TSPO ligand Ro5-4864 leads to decreased amyloid load in the hippocampus, decreased glial activation, and ameliorated behavioral deficits in the 3xTgAD mouse model of AD [95]. Moreover, TSPO overexpression in the CA1 region of the hippocampus improved LPS-induced cognitive impairments in mice [96]. Because TSPO is increased on activated microglia [92, 93], it is not surprising that TSPO is increased on microglia in our AD model following IL-1β overexpression. However, because TSPO was particularly increased in MX04^+^ microglia, dissecting the role of TSPO in facilitating amyloid beta clearance will be important to address in future studies.

## Conclusion

In conclusion, we demonstrated that microglia are activated, proliferate, and are the main cell type associated with Aβ removal following sustained IL-1β expression. Our studies also suggest that IL-1β induces changes in the expression of genes related to immune responses, proliferation, cytokine signaling, and AD pathology in microglia, and that microglia may facilitate Aβ clearance either directly by local self-proliferation and/or by the interplay of these gene expression changes. Future studies will carefully evaluate the implications of these gene expression changes in microglia following IL-1β overexpression and their contribution to Aβ plaque clearance. Understanding the interplay of these gene expression changes on microglia in the context of chronic neuroinflammation will have implications for future therapies targeting immune-related pathways in Alzheimer’s disease.

## Supplementary information

**Additional file 1. **Sustained hippocampal expression of IL-1β reduces amyloid plaque burden in both female and male APP/PS1 mice. (A) Quantification of 6E10 (left) and Congo Red (right) staining displayed as percent area of hippocampus covered by amyloid plaques in APP/PS1 mice treated with rAAV2-Phe or rAAV2-IL-1β. n = 4-6 mice per group. Data displayed as mean ± SEM, two-way ANOVA, **p < 0.005, ***p < 0.0005, ****p < 0.0001.

**Additional file 2. **IL-1β drives reductions of existing amyloid plaques in APP/PS1 mice. (A) Schematic of methoxy-X04 injections in APP/PS1 mice and viral vector transduction. Seven month-old APP/PS1 mice were injected with MX04 to label pre-existing amyloid plaques in vivo prior to AAV2 transduction. Three days following MX04 injection, mice were transduced in the hippocampus with rAAV2-Phe or rAAV2-IL-1β. Brains were collected three weeks post-AAV2 transduction and processed for IHC analysis of MX04 staining of amyloid plaques. (B) Quantification of percent area of MX04 staining of amyloid plaques and plaque counts in APP/PS1 mice treated with rAAV2-Phe or rAAV2- IL-1β. n = 3 mice. Data displayed as mean ± SEM, unpaired t-test, *p < 0.05, **p < 0.005.

**Additional file 3. **IL-1β induces recruitment of CD45^hi^ myeloid cells to the hippocampus in APP/PS1 mice. Total number (left) and percentage (right) of recruited myeloid cells (CD45^hi^) in hippocampus of APP/PS1 mice treated with rAAV2-Phe or rAAV2-IL-1β. n = 9-12 per group. Data displayed as mean ± SEM, multiple t-tests corrected for multiple comparisons using the Holm-Sidak method, **p < 0.005, ****p < 0.0001.

**Additional file 4.** Absolute numbers of MX04^+^ cells.

**Additional file 5.** RNAseq data.

## Data Availability

All RNA-sequencing data files were submitted to the Gene Expression Omnibus (GEO) database under accession number GSE113539.

## Abbreviations

AD: Alzheimer’s disease
APP: amyloid precursor protein
APP/PS1: APPswe/PS1dE9
Aβ: amyloid beta
BMD: bone marrow-derived
BrdU: bromodeoxyuridine
FACS: fluorescence-activated cell sorting
GFAP: glial fibrillary acidic protein
Iba1: ionized calcium binding adaptor molecule 1
IL-1β: interleukin-1 beta
MX04: methoxy-X04
PI: propidium iodide
rAAV2: recombinant adeno-associated virus serotype 2
