# Supplementary figures and images for: IL-1β-driven amyloid plaque clearance is associated with an expansion of transcriptionally reprogrammed microglia
Source: J Neuroinflammation. 2019 Dec 10;16:261. doi: 10.1186/s12974-019-1645-7 (PMC6902486; doi:10.1186/s12974-019-1645-7)

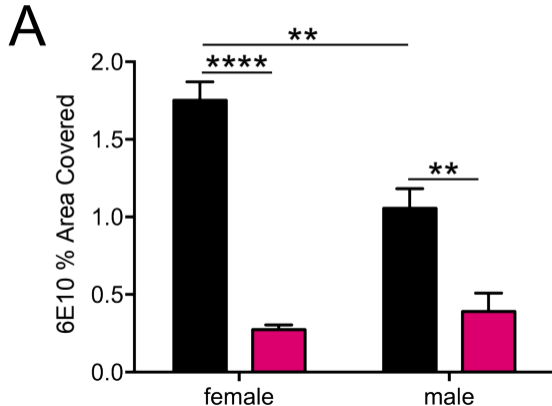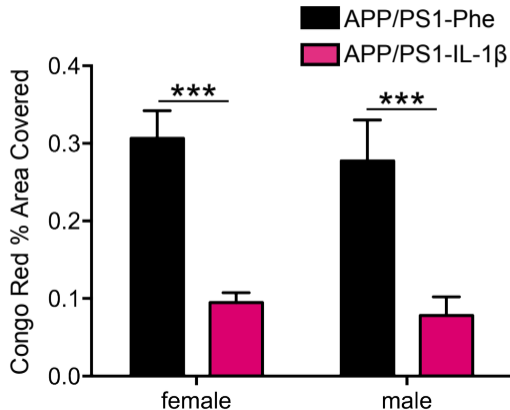

Supplement: Supplementary file 1 — Additional file 1. Sustained hippocampal expression of IL-1β reduces amyloid plaque burden in both female and male APP/PS1 mice. (A) Quantification of 6E10 (left) and Congo Red (right) staining displayed as percent area of hippocampus covered by amyloid plaques in APP/PS1 mice treated with rAAV2-Phe or rAAV2-IL-1β. n = 4-6 mice per group. Data displayed as mean ± SEM, two-way ANOVA, **p < 0.005, ***p < 0.0005, ****p < 0.0001. [file 12974_2019_1645_MOESM1_ESM.pdf]

A

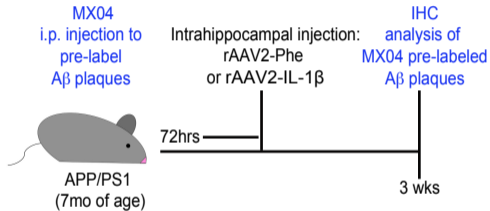

B

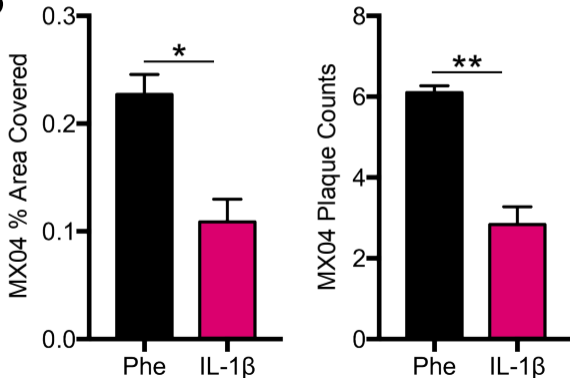

Supplement: Supplementary file 2 — Additional file 2. IL-1β drives reductions of existing amyloid plaques in APP/PS1 mice. (A) Schematic of methoxy-X04 injections in APP/PS1 mice and viral vector transduction. Seven month-old APP/PS1 mice were injected with MX04 to label pre-existing amyloid plaques in vivo prior to AAV2 transduction. Three days following MX04 injection, mice were transduced in the hippocampus with rAAV2-Phe or rAAV2-IL-1β. Brains were collected three weeks post-AAV2 transduction and processed for IHC analysis of MX04 staining of amyloid plaques. (B) Quantification of percent area of MX04 staining of amyloid plaques and plaque counts in APP/PS1 mice treated with rAAV2-Phe or rAAV2- IL-1β. n = 3 mice. Data displayed as mean ± SEM, unpaired t-test, *p < 0.05, **p < 0.005. [file 12974_2019_1645_MOESM2_ESM.pdf]

A

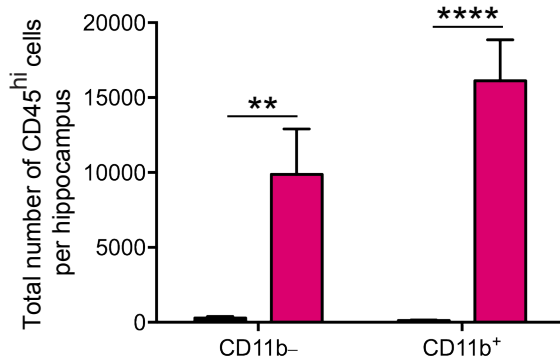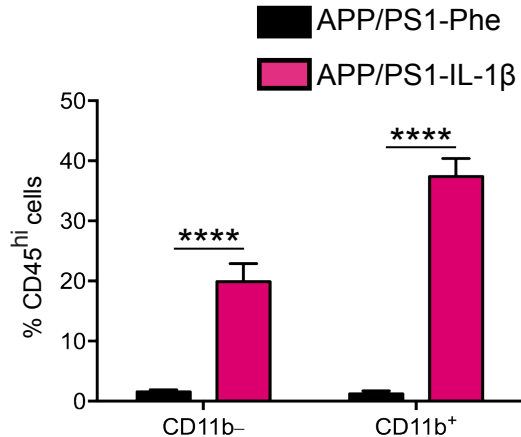

Supplement: Supplementary file 3 — Additional file 3. IL-1β induces recruitment of CD45hi myeloid cells to the hippocampus in APP/PS1 mice. Total number (left) and percentage (right) of recruited myeloid cells (CD45hi) in hippocampus of APP/PS1 mice treated with rAAV2-Phe or rAAV2-IL-1β. n = 9-12 per group. Data displayed as mean ± SEM, multiple t-tests corrected for multiple comparisons using the Holm-Sidak method, **p < 0.005, ****p < 0.0001. [file 12974_2019_1645_MOESM3_ESM.pdf]
