# Supplementary material for: IL-1β-driven amyloid plaque clearance is associated with an expansion of transcriptionally reprogrammed microglia
Source: J Neuroinflammation. 2019 Dec 10;16:261. doi: 10.1186/s12974-019-1645-7 (PMC6902486; doi:10.1186/s12974-019-1645-7)
Supplement: Supplementary file 4 — Additional file 4. Absolute numbers of MX04+ cells. [file 12974_2019_1645_MOESM4_ESM.docx]

**Additional File 4: Absolute Numbers of MX04^+^ Cells**

| **Cell Type**  **(Absolute Numbers)** | **APP/PS1-Phe**  (n = 9) | **APP/PS1-IL-1β**  (n = 10) | ***p*-value** |
| --- | --- | --- | --- |
| CD45^-^CD11b^-^ | 60.83 ± 13.27 | 100.4 ± 23.44 | 0.0860 |
| CD45^hi^CD11b^-^ | 0 ± 0 | 27.35 ± 19.47 | 0.1007 |
| CD45^hi^CD11b^+^ | 3.33 ± 1.58 | 89.28 ± 37.24 | 0.0218 |
| CD45^lo^CD11b^+^ | 464.9 ± 88.72 | 785.5 ± 124.9 | 0.0281 |

Data are presented as mean ± SEM.
